# The Influence of Sodium Humate on the Biosynthesis and Contents of Flavonoid Constituents in Lemons

**DOI:** 10.3390/plants13202888

**Published:** 2024-10-15

**Authors:** Nianao Xu, Fan Yang, Weifeng Dai, Cheng Yuan, Jinxue Li, Hanqi Zhang, Youdi Ren, Mi Zhang

**Affiliations:** 1 Faculty of Life Science and Technology, Kunming University of Science and Technology, Kunming 650500, China; qazwsxxna@163.com (N.X.); dwflove@163.com (W.D.); chengyuan@kust.edu.cn (C.Y.); qazwsxzhq@163.com (H.Z.); deeren5464@163.com (Y.R.); 2 Institute of Tropical and Subtropical Cash Crops, Yunnan Academy of Agricultural Sciences, Ruili 678600, China; yflyxy@126.com (F.Y.); ljxue810@163.com (J.L.)

**Keywords:** sodium humate, flavonoid, action mechanism, lemon, HCT, F5H, phenylalanine pathway, humic acid

## Abstract

Sodium humate (SH) is the sodium salt of humic acid. Our previous research has demonstrated that SH has the ability to enhance the levels of total flavonoids in various parts of lemons, including the leaves, peels, pulps, and seeds, thereby improving the quality of lemons. In the current study, the regulation effect of SH on the biosynthesis and content of lemon flavonoid compounds was examined using transcriptome sequencing technology and flavonoid metabolomic analysis. Following SH treatment, the transcriptome sequencing analysis revealed 320 differentially expressed genes (DEGs) between samples treated with SH and control (CK) samples, some of which were associated with the phenylalanine pathway by KEGG annotation analysis. The levels of seven flavonoid compounds identified in lemon peels were observed to increase, and eriocitrin and isoorientin were identified as differential metabolites (DMs, VIP > 1) using OPLS-DA analysis. The integrated analysis of transcriptomics and flavonoid metabolomics indicates that SH treatment induces alterations in gene expression and metabolite levels related to flavonoid synthesis. Specifically, SH influences flavonoid biosynthesis by modulating the activity of key enzymes in the phenylalanine pathway, including HCT (O-hydroxycinnamoyltransferase) and F5H (ferulate-5-hydroxylase).

## 1. Introduction

Secondary metabolism in plants is the metabolic processes that occur within the plant, aside from basic metabolism (such as energy production and fundamental life processes). It plays a crucial chemo-ecological role in the interactions between plants, between plants and microorganisms, and between plants and insects [1,2]. Secondary metabolites in plants comprise a class of small organic molecules synthesized through secondary metabolism, including flavonoids, terpenes, and alkaloids, among others [3]. These compounds perform vital functions by regulating growth, enhancing resistance to diseases and herbivores, attracting pollinators, and supporting overall plant survival and reproduction [4,5]. Additionally, these substances possess unique pharmacological properties [6], contributing significantly to the quality and economic value of various plant products including crops, herbal medicines, and traditional Chinese medicines [7].

The complex array of secondary metabolites and their intricate metabolic pathways are influenced by specific environmental and temporal conditions, occurring in particular species, organs, or tissues [8]. This often leads to relatively low levels of these compounds in plants under natural conditions. Numerous studies suggest that plant metabolites are the ultimate response of biological systems to genetic or environmental alterations [9]. Various environmental factors such as light, temperature, drought, salinity, nutrients, pathogens, and insects can impact the types and concentrations of plant metabolites [10,11,12]. Through the use of advanced analytical techniques like transcriptome sequencing technology, genome sequencing techniques, and liquid chromatography-mass spectrometry (LC-MS), researchers can observe dynamic changes in multiple genes and metabolites before and after disturbances or stimuli. This process facilitates the identification of differentially expressed genes and metabolites, serving as a valuable tool for exploring the biosynthesis, functions, regulatory mechanisms, ecological effects, etc. of secondary metabolites.

*Citrus limon* (L.) Burm. f. is a widely recognized valuable economic crop with culinary and medicinal applications. Lemon branches, leaves, flowers, and fruits are not only rich in essential vitamins and trace elements needed by the human body, but they also contain flavonoids and terpenes, which have various biological functions. These constituents not only contribute to the distinctive flavor and taste of lemons but also play vital roles in their nutritional, cosmetic, health, and medicinal benefits [13,14,15]. Research on flavonoid compounds present in lemons, such as hesperidin, naringin, neohesperidin, and rutin, has shown varied physiological effects, including anti-inflammatory, anti-hypertensive, and cholesterol-lowering properties [16,17,18,19]. As a result, flavonoids have become key components defining lemon quality.

Sodium humate (SH) is the sodium salt of humic acid, which is widely used in agriculture to improve soil quality, enhance soil structure, promote plant growth, increase crop yield, and promote the synthesis of secondary metabolites in plants. In our previous investigation, we have found that spraying C. limon with humic/fulvic acid fertilizers can enhance the levels of vitamin C, total acid, total sugar, total flavonoids, and phenols in its fruits [20], and spraying SH on leaves with the optimal application dosage of 5 g/L can increase the total flavonoid content in lemon leaves [21]. In the current study, we investigated the mechanism of action of sodium humate on flavonoids in lemons using transcriptome sequencing technology and metabolomic analysis. Through the integration of transcriptomic and flavonoid metabolomic analyses, we initially elucidated the impact of SH treatment on flavonoid biosynthesis.

## 2. Results

### 2.1. Analysis of the Differentially Expressed Genes (DEGs) between the Distilled-Water-Treated and Sodium-Humate-Treated Groups

**Figure 1:**
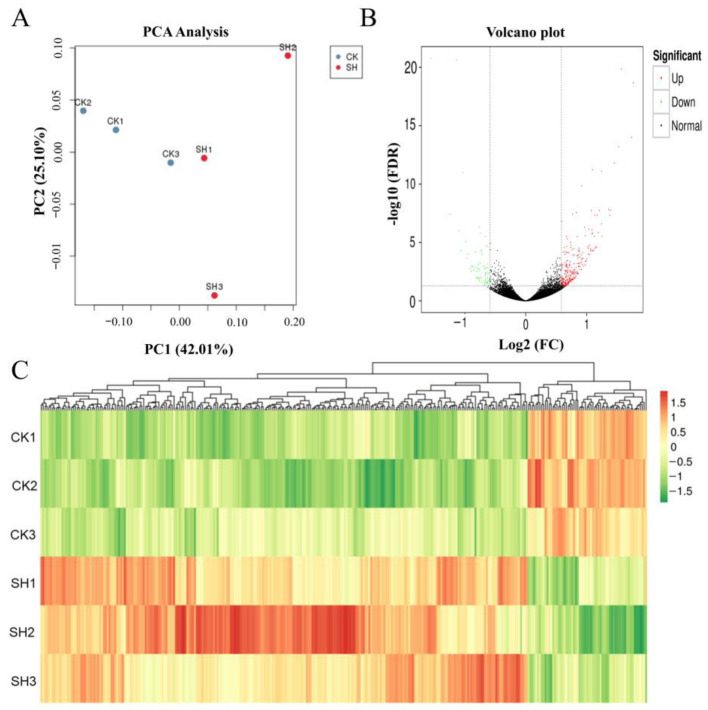
Overview of the effects of SH treatment on lemon gene expression (total RNA was extracted from lemon leaves). (**A**) Principal component analysis (PCA) of the transcripts of the lemon treated with SH. (**B**) The differential gene volcano map. Green represents gene downregulation, red represents gene upregulation, and black dots represent genes with no significant expression difference. (**C**) Cluster plot of differentially expressed gene expression patterns. Different rows in the figure represent different samples, and different columns represent different genes. Colors represent the log values of the bottom 2 for FPKM in the sample.

The differentially expressed genes (DEGs) between the distilled-water-treated (CK) and SH-treated (SH) groups were analyzed using transcriptome sequencing technology. Each group consisted of three samples. Following quality control, 189,594,815 raw reads were obtained, totaling 56.4 G of clean data (Table S1). The 44,533 unigenes obtained were compared with the KEGG database using BLAST, and 33.8% of the total genes were successfully annotated (Table S2). The gene expression levels of the samples were visualized through Principal Component Analysis (PCA) (Figure 1A), revealing that SH induced alterations in the genes of lemon. Quantitative statistics by differentially expressed genes revealed 320 differentially expressed genes between SH and CK samples, of which 257 were upregulated and 63 were downregulated (Figure 1B). After alignment with various databases, cluster analysis of differentially expressed genes was performed, and a total of 307 genes were annotated between CK and SH groups, with 106 annotated by KEGG. As shown in Figure 1C, the annotated genes between CK and SH groups were mainly concentrated on the upregulated genes.

**Figure 2:**
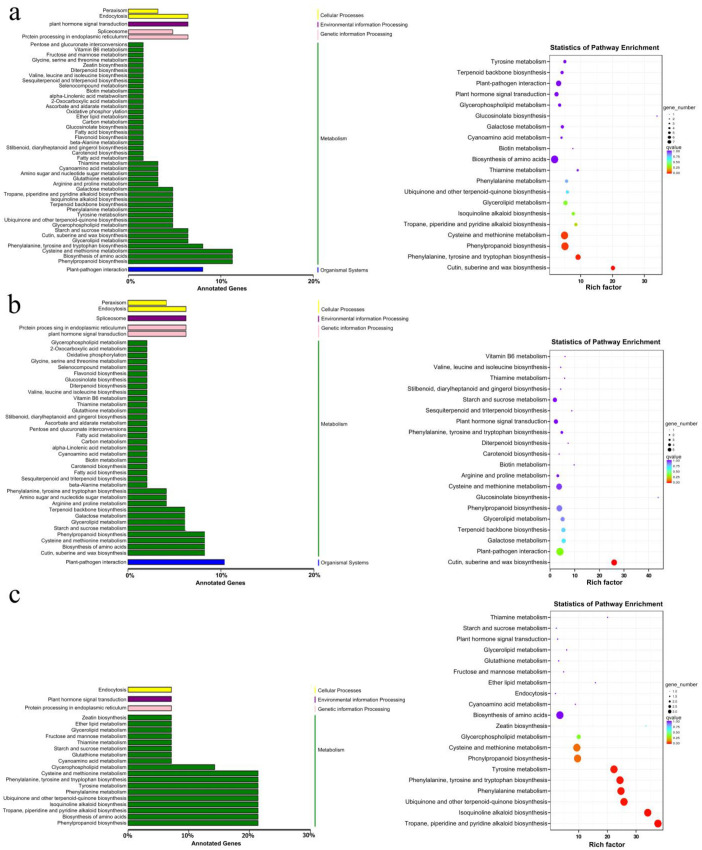
The KEGG classification map of differentially expressed genes versus the scatter plot of pathway enrichment of KEGG genes represents the KEGG metabolic pathways. The x-axis indicates the number of genes annotated to the pathway and the proportion of this number compared to the total number of annotated genes. (**a**) Total differentially expressed genes. (**b**) Upregulated genes. (**c**) Downregulated genes.

The KEGG annotation analysis of DEGs between the CK and SH groups, as depicted in Figure 2, reveals that the upregulated genes are primarily associated with plant–pathogen interactions, amino acid biosynthesis, phenylpropane biosynthesis, and the metabolism of cysteine, methionine, cutin, suberine, and waxes. Conversely, the downregulated genes are mainly involved in isoquinoline alkaloid biosynthesis, phenylpropane biosynthesis, phenylalanine metabolism, amino acid biosynthesis, tropane, piperidine, and pyridine alkaloid biosynthesis, as well as ubiquinone and other terpenoid-quinone biosynthesis, tyrosine metabolism, phenylalanine, tyrosine, tryptophan biosynthesis, cysteine, and methionine metabolism.

### 2.2. Qualitative and Quantitative Analysis on Flavonoids from the Lemon Peels

Lemon peel is the part of the lemon that contains a higher content of flavonoids. To investigate the impact of SH on lemon flavonoids, lemon fruits treated with SH or distilled water were collected during three different harvest periods. The flavonoid monomers from the peels were then qualitatively and quantitatively analyzed using HPLC-DAD-MS.

**Figure 3:**
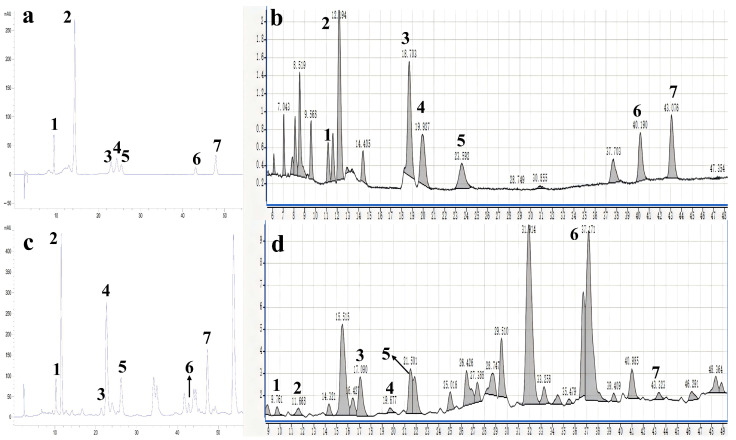
HPLC-DAD-MS chromatogram of a standard prepared with standard compounds and samples prepared from lemon peels: (**a**) HPLC-DAD chromatogram of the standard; UV detection (λ = 330 nm). (**b**) The HPLC-MS analysis of the standard in the negative electrospray mode. (**c**) HPLC-DAD chromatogram of samples; UV detection (λ = 330 nm). (**d**) HPLC-MS analysis of samples in the negative electrospray mode. 1: vicenin-2; 2: isoorientin; 3: eriocitrin; 4: vitexin; 5: rutin; 6: narcissoside; 7: diosmin.

Seven specific flavonoid compounds were identified as vicenin-2 (**1**), isoorientin (**2**), eriocitrin (**3**), vitexin (**4**), rutin (**5**), narcissoside (**6**), and diosmin (**7**) through the analysis of chromatographic peak samples, by comparing retention times, UV absorption properties, and MS data (Figure 3, Figure S1, Table S3) from standard and reference reports.

**Figure 4:**
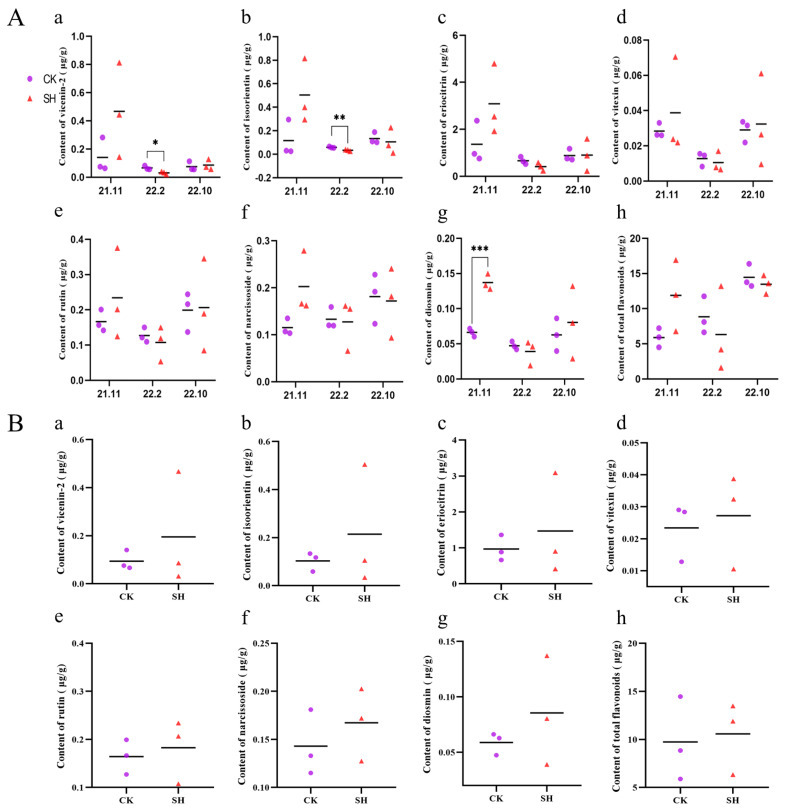
Contents of flavonoids in the samples prepared from lemon peels determined by HPLC-DAD. (**A**) The flavonoid content at each harvest period. (**B**) The flavonoid content during three harvest periods. (**a**) Vicenin-2; (**b**) isoorientin; (**c**) eriocitrin; (**d**) vitexin; (**e**) rutin; (**f**) narcissoside; (**g**) diosmin; (**h**) total flavonoids. Data are presented as means ± standard deviation (*n* = 3). *** *p* < 0.001, ** *p* < 0.01, * *p* < 0.05, vs. the control group.

Moreover, the content of the seven flavonoids was determined using the standard curve method in combination with the HPLC-DAD technique, while the total flavonoids were measured using the established standard curve method combined with the UV technique. The results (Figure 4 and Table S4) revealed that the influence of SH on the seven flavonoid components and total flavonoids in the peel varies among different harvesting periods (Figure 4A). Nevertheless, compared to the control group, SH increases the levels of the seven flavonoid compounds and total flavonoids in lemon peel when considering the three harvesting periods as a whole (Figure 4B).

### 2.3. Method Evaluation

In the stability test, the RSD values for vicenin-2, isoorientin, eriocitrin, vitexin, rutin, narcissoside, diosmin, and total flavonoids at specific time points were 0.32%, 0.60%, 0.54%, 1.97%, 0.54%, 1.02%, 2.30%, and 1.88%, respectively (Table S5). The RSD values for all seven flavonoids and total flavonoids were below 3%, indicating the excellent stability of the method.

In the reproducibility test, the RSD values for vicenin-2, isoorientin, eriocitrin, vitexin, rutin, narcissoside, diosmin, and total flavonoids were 0.39%, 0.46%, 1.04%, 1.02%, 0.59%, 1.90%, 0.71%, and 1.20%, respectively (Table S5). The RSD values of all compounds were below 3%, illustrating the good reproducibility of the method.

The precision test included vicenin-2, isoorientin, eriocitrin, vitexin, rutin, narcissoside, diosmin, and total flavonoids, with RSD values of 0.58%, 0.49%, 0.57%, 1.14%, 0.56%, 1.00%, 0.50%, and 0.11%, respectively (Table S5), demonstrating the accuracy of the method.

In the recovery experiments, the calculated recovery rates for vicenin-2, isoorientin, eriocitrin, vitexin, rutin, narcissoside, diosmin, and total flavonoids were 114.18%, 96.08%, 105.14%, 112.71%, 91.35%, 107.49%, 88.91%, and 93.50%, respectively (Table S5), indicating the method’s suitability for target flavonoids.

### 2.4. Comprehensive Analysis of Transcriptomics and Metabolomics Data on Flavonoid Biosynthesis in Lemon

**Figure 5:**
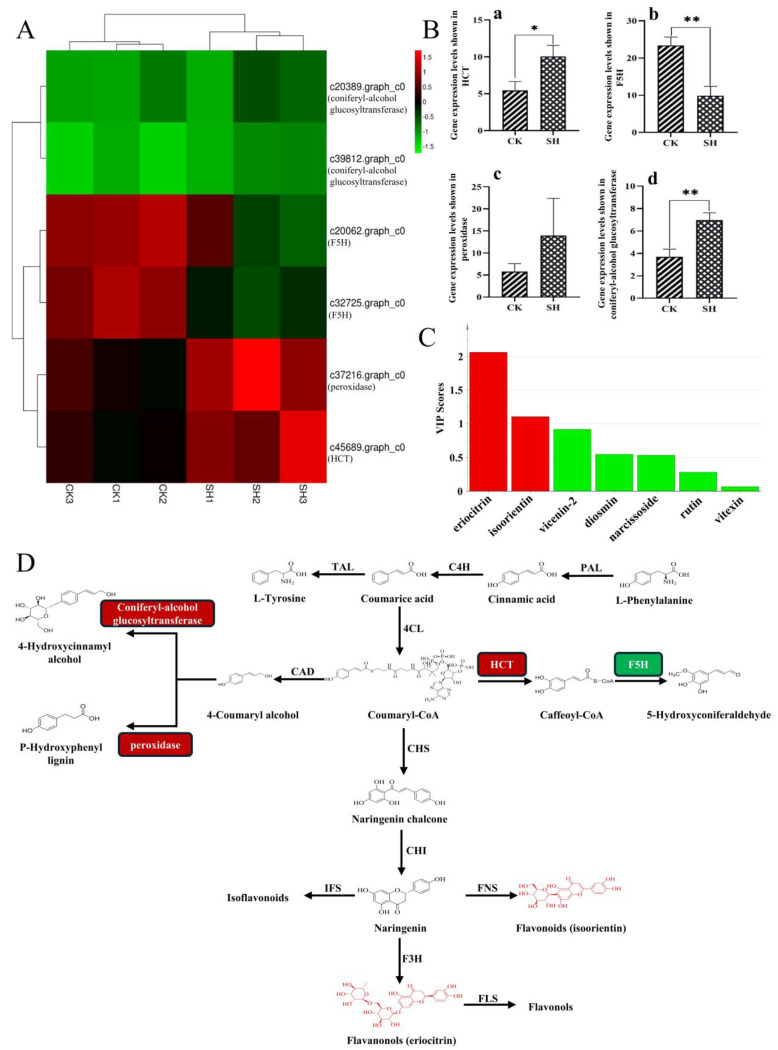
Overview of the relationship between DEGs and DMs in the lemon treated by SH. (**A**) Cluster plot of DEGs enriched in phenylpropane pathway. Different rows in the figure represent different samples, and different columns represent different genes. (**B**) Analysis of the gene expression levels of the four enzymes in the phenylalanine pathway (*n* = 3). ** *p* < 0.01, * *p* < 0.05, vs. the control group; (**a**): HCT (O-hydroxycinnamoyltransferase), (**b**): F5H (ferulate-5-hydroxylase), (**c**): peroxidase, (**d**): coniferyl-alcohol glucosyltransferase. (**C**) VIP plot of differential metabolites of flavonoids in lemon analyzed by OPLS-DA. (Red indicates differential metabolites with VIP > 1). (**D**) Overview of flavonoid biosynthesis via phenylalanine pathway. PAL: Phenylalanine ammonia-lyase; TAL: Tyrosine ammonia-lyase; C4H: Cinnamic acid 4-hydroxylase; 4CL: 4-Coumaric acid-CoA ligase; F5H: ferulate-5-hydroxylase; HCT: O-hydroxycinnamoyltransferase; CAD: dehydrogenase; CHS: Chalcone synthase; CHI: Chalcone isomerase; FNS: Flavone synthase; IFS: Isoflavone synthase; F3H: Flavanone-3-hydroxylase; FLS: Flavonol synthase.

The biosynthesis of flavonoid compounds in plants occurs via the phenylalanine pathway. To investigate the effect of sodium humate (SH) on the biosynthesis of flavonoid compounds in lemon, the differentially expressed genes (DEGs) involved in the phenylalanine pathway from the KEGG results was analyzed and shown in Figure 5A. DEGs (c20062.graph_c0, c20389.graph_c0, c32725.graph_c0, c37216.graph_c0, c39812.graph_c0, c45689.graph_c0) encoding 4 key enzymes were involved in the biosynthesis of phenylalanine pathway (Table S6), including O-hydroxycinnamoyltransferase (HCT), ferulate-5-hydroxylase (F5H), glucosyltransferase and peroxidase. In comparison to the control group, the gene expression levels of HCT, glucosyltransferase, and peroxidase were higher in the SH-treated group, while the expression level of F5H was lower in the SH-treated group (Figure 5B).

In metabolomics research, statistical analyses (such as PCA, OPLS-DA analysis, etc.) are used to identify metabolites that show significant differences in concentration between specific sample groups. Such metabolites that show significant differences are termed differential metabolites. Based on the content determination results obtained through HPLC-DAD-MS, an OPLS-DA model was developed to examine the differential metabolites of flavonoids. Figure 5C illustrates that two flavonoids (eriocitrin and isoorientin) with VIP scores greater than 1 were identified as the differential metabolites (DMs) in the SH and CK groups.

Flavonoid biosynthesis necessitates the participation of both biosynthetic and modifying enzymes, as well as certain regulatory factors and transporters. Based on our results, the DEGs and DMs involved in the phenylalanine pathway were mapped onto the flavonoid biosynthesis pathway (Figure 5D). The results indicate that HCT, F5H, glucosyltransferase, and peroxidase do not directly participate in the conversion of flavonoid compounds from the intermediate coumaryl-CoA; however, they still influence the biosynthesis of flavonoid compounds through the phenylalanine pathway.

## 3. Discussion

Flavonoids are common secondary metabolites in plants. Through detailed exploration of their structure and activities, these compounds have demonstrated significant potential for various applications. For example, the flavonoid components in lemons can be applied in functional foods, daily chemical products, health care products, pharmaceuticals, and others. Thus, in addition to traditional Chinese herbal medicine, an increasing number of economic crops are also using the content of flavonoid components as a quality indicator that requires testing [22]. To investigate the mechanisms of action of SH on lemon flavonoids, this study explored the influence of SH on the biosynthesis and content of lemon flavonoid compounds using transcriptome sequencing technology and flavonoid metabolomic analysis.

Research indicates that the biosynthesis of flavonoids commences with the transformation of phenylalanine into coumaroyl-CoA via the phenylalanine metabolic pathway, which then combines with malonyl-CoA produced during sugar metabolism to form chalcone. Subsequent biological modifications convert chalcone into dihydroflavonoids. Ultimately, dihydroflavonoids undergo further bio-modifications to produce a range of compounds such as flavonoids, isoflavonoids, flavonols, flavanols, and anthocyanins [23,24,25]. In this biosynthetic pathway, many enzymes play a crucial role in accumulation of flavonoids, such as PAL, 4CL, C4H, CHS, CHI, FLS, F3H, DFR, etc. The activity of these enzymes, as well as their molecular synthesis levels, are influenced by environmental factors such as light, temperature, moisture, and minerals [26,27]. Through transcriptome sequencing, 320 differentially expressed genes (DEGs) between the SH treatment group and control group were identified. Subsequently, these DEGs (c20062.graph_c0, c20389.graph_c0, c32725.graph_c0, c37216.graph_c0, c39812.graph_c0, c45689.graph_c0) were annotated to phenylalanine pathway via KEGG functional enrichment analysis, including HCT, F5H, glucosyltransferase and peroxidase. Previous investigations have shown that miRNAs can regulate target genes directly or indirectly through transcription factors, thereby helping to maintain the balance of carbon flow between flavonoids and lignin. The genes of HCT and F5H have demonstrated potential roles in inhibiting lignin synthesis [28,29]. Notably, in *Arabidopsis*, HCT is known to inhibit lignin synthesis, thereby redirecting metabolic flux towards flavonoids via chalcone synthase activity [30]. In the leaves of *Salvia miltiorrhiza*, higher expression levels of HCT and increased concentrations of flavonoids were observed in the purple phenotype compared to the green phenotype. This finding suggests that HCT may act as a positive regulator of flavonoid biosynthesis, thereby influencing the coloration of the organs and tissues in *S. miltiorrhiza* [31]. In our study, the expression levels of HCT and F5H exhibited significant differences between the SH-treated group and the control group. This evidence suggests that SH primarily influences the biosynthesis of flavonoid compounds by regulating HCT (O-hydroxycinnamoyltransferase) and F5H (ferulate-5-hydroxylase), which are critical enzymes in the phenylpropanoid pathway responsible for flavonoid biosynthesis.

Lemon fruit is the main edible part of the lemon. Generally, the flavonoid content varies significantly among the different tissues of lemons and the flavonoid content in the lemon fruit is higher than in other parts [32]. In this study, lemon peels from three distinct harvesting periods were collected and both qualitative and quantitative analyses of total flavonoids and seven flavonoid compounds were carried out. The results demonstrated that the flavonoid content varied to different extents across the three harvesting periods when compared to the control group. Nevertheless, comprehensive analysis revealed that the flavonoid components in the treatment group were elevated compared to the control group. Through OPLS-DA analysis, two flavonoids were identified as differential metabolites (VIP > 1), which was consistent with the findings from the transcriptome sequencing analysis and indicated the influence of SH on flavonoid biosynthesis.

## 4. Materials and Methods

### 4.1. Materials and Reagents

Plant materials (“Yunning No. 1”, an improved variant of Eureka lemon) were collected from 4–5-year-old lemon trees in the Lemon Base of Tropical and Subtropical Cash Crops of Yunnan Academy of Agricultural Sciences (Ruili, China). Sodium humate was provided by Shangcheng Biotechnology Co., Ltd. (Yuxi, China). The standards of vicenin-2 (>98%), isoorientin (>98%), eriocitrin (>98%), vitexin (>98%), rutin (>98%), narcissoside (>98%), diosmin (>98%) and other chemical products were purchased from Chengdu Yirui Company (Chengdu, China).

### 4.2. Processing Method and Sampling Method

Sodium humate (SH) was dissolved in distilled water to produce a 5 g/L solution. In the SH-treated group (10 plants per group, 3 parallel groups), each plant was sprayed with 5 L of the 5 g/L SH solution once on the 1st of each month from October 2020 to November 2022, following the same spraying schedule as the distilled water treatment group. Lemon leaves were randomly collected for transcriptome sequencing analysis on 30 November 2020. The peels from lemon fruits were randomly selected on 30 November 2021, 30 February 2022, and 30 October 2022.

### 4.3. Transcriptome Sequencing and Data Analysis

To investigate the effect of various treatments on the transcript levels in lemon, total RNA was extracted from lemon leaves preserved at −80 °C, with samples taken in triplicate for each treatment group. Library construction and quality assessment were conducted using methods provided by Baimak Company (Chengdu, China). To ensure the quality and reliability of data analysis, raw data were filtered to remove low-quality reads (Q30 > 0.85), reads with more than 10% unknown bases, and ribosomal RNA content exceeding 10%. The Trinity Software (v2.5.1) was employed for transcript preparation. Unigene sequences were aligned with the KEGG database using BLAST software (v2.2.31), and KOBAS v2.0 was utilized to acquire KEGG Orthology results for Unigene, providing annotation information. Differential expression analysis was performed between sample groups using DESeq2 (v1.6.3) to identify differentially expressed gene sets under the two conditions. The resulting *p*-values were adjusted with the Benjamini–Hochberg method, yielding corrected *p*-values and the False Discovery Rate (FDR). Genes with an adjusted FDR < 0.05 and a Fold Change (FC) ≥ 1.5 were classified as differentially expressed. Annotation and enrichment analyses of the differentially expressed genes were conducted using KEGG.

### 4.4. Flavonoid Metabolite Analysis

To prepare the lemon peel extract, the fresh lemon peel was cut into small pieces and dried using a freeze dryer. Then, 10 g of the dried peel was placed in a 250 mL tapered flask. Following this, 200 mL of petroleum ether was added and sonicated for 45 min (this process was repeated 3 times). Subsequently, the petroleum ether volatilized, and 200 mL of 80% methanol was added and sonicated for 40 min (this step was repeated 2 times). The filtrate from both steps was combined, the solvent was concentrated, and the extracted peel was obtained.

To determine the total flavonoid content, 2.0 mg of the extract was dissolved in 10 mL of 80% methanol to prepare the sample, and its UV absorbance was measured at 510 nm using NaNO_2_-Al(NO_3_)_3_-NaOH spectrophotometry. The measurement was conducted on T6 new Century type ultraviolet visible spectrophotometer (Beijing General General Instrument Co., Ltd., Beijing, China). The total flavonoid content in the sample was calculated using the standard curve method of rutin. The standard stock solution was prepared by dissolving 0.01 g of rutin in 100 mL of 80% methanol. This stock solution was then diluted to various concentrations to create a standard solution, which was analyzed at 510 nm to generate a standard curve using UV spectrophotometry. The equation for total flavonoids, along with correlation coefficients (R^2^) exceeding 0.999, was documented in Table S5. These data were used to determine the concentration of total flavonoids in the samples.

The qualitative and quantitative analysis of flavonoid compounds was performed using the HPLC-DAD-MS method. Initially, 0.2 g of the extract was dissolved in 2 mL of 80% methanol and filtered through a 0.45 μm filter into an SPE column (Waters Sep-Pak C18, 20 cc/5 g, Milford, MA, USA). Subsequently, the SPE column was eluted with 375 mL of 18% methanol, followed by 80% methanol to yield 250 mL of eluate. The eluate was then concentrated to 2 mL under reduced pressure. The samples were stored in a refrigerator at 4 °C for future analysis.

For qualitative analysis of flavonoid compounds, an Agilent 1260 HPLC system coupled with an Agilent 6530B Q-TOF mass spectrometer (Agilent, Santa Clara, CA, USA) operated in negative electrospray ionization (ESI) mode was utilized. The following analysis conditions were employed: solvent A: 0.05% formic acid in water (*v*/*v*); solvent B: acetonitrile. An Agilent Eclipse XDB-C18 (4.6 mm, 5 μm) column was utilized. The gradient conditions were as follows: 0–3 min, 10–15% B; 3–28 min, 15% B; 28–38 min, 15–18% B; 38–58 min, 18–21% B (*v*/*v*). The injection volume was 20 μL, the flow rate was 1 mL/min, the detection wavelength was set at 330 nm, and the column temperature was maintained at 25 °C. The ion source spray voltage was 3500 V, with a dry gas flow rate of 10 L/min, Nebulizer at 30 pins, Fragmentor at 175 V, and the ion source temperature set to 325 °C.

The quantitative analysis of flavonoid compounds was conducted using the standard curve method established by HPLC-DAD. Stock solutions of 0.2 mg/mL diosmin, 1 mg/mL isoorientin, 0.1 mg/mL narcissoside, 0.2 mg/mL rutin, 1 mg/mL eriocitrin, 0.2 mg/mL vitexin, and 0.2 mg/mL vicenin-2 were prepared and subsequently diluted to various concentrations for the standard solution. These solutions were then analyzed via the HPLC-DAD under the aforementioned conditions.

The equations for each flavonoid are listed in (Table S5) with correlation coefficients (R^2^) greater than 0.999, indicating a good fit, based on which the concentration of each flavonoid was obtained by measuring the content of each flavonoid in the sample.

The equations for each flavonoid are listed in Table S5, with correlation coefficients (R^2^) exceeding 0.999, indicating a strong fit. These equations were used to calculate the concentration of each flavonoid by measuring the absorbance peak areas detected by HPLC-DAD.

In the above-mentioned analysis, six quantities of each flavonoid sample were measured to calculate the relative standard deviation (RSD). Precision experiments were carried out on the rutin standard and each described flavonoid analytically. The stability experiment determined the RSD of both the total and each flavonoid sample at various times (9:00 daily for five days). For the recovery test, the standard solution was mixed with the sample solution at an approximate 1:1 ratio (*v*/*v*), and the RSD of both the total and each flavonoid sample was calculated five times using the same analytical method.

### 4.5. Data Analysis

The data were analyzed and visualized using GraphPad Prism 8.0.1. Statistical significance was determined with the Student’s *t*-test. All experiments were conducted with a minimum of three replicates.

## 5. Conclusions

Sodium humate (SH) treatment induces alterations in gene expression and metabolite levels related to flavonoid biosynthesis. It influences flavonoid biosynthesis by modulating the activity of key enzymes in the phenylalanine pathway, including HCT (O-hydroxycinnamoyltransferase) and F5H (ferulate-5-hydroxylase).

## Data Availability

Data are contained within the article and Supplementary Materials.

## Supplementary Materials

The following supporting information can be downloaded at: https://www.mdpi.com/article/10.3390/plants13202888/s1, Figure S1: The ultraviolet-visible (UV) and mass (MS) spectra of determined flavonoids in standards and samples prepared from lemon peels; Table S1: Summary of sequencing data of lemon leaves and information of clean reads; Table S2: Unigene annotated statistics; Table S3: Characterization of seven flavonoids from the extract of lemon peels by high performance liquid chromatography-diode array detector/electrospray ionization mass spectrometry (HPLC-DAD/ESIMS); Table S4: The contents of 7 flavonoids in samples extracted from lemon peels treated or untreated with sodium humate (SH) by high performance liquid chromatography-diode array detector (HPLC-DAD); Table S5: Linear regression, equation precision of 8 flavonoids from extract of lemon peels treated sodium humate (SH), the RSD values (%) of precision, repeatability, stability and recovery rate; Table S6: Analysis of proteins and expression of differential genes in the phenylpropane metabolic pathway.
