# Supplementary material for: The Influence of Sodium Humate on the Biosynthesis and Contents of Flavonoid Constituents in Lemons
Source: Plants (Basel). 2024 Oct 15;13(20):2888. doi: 10.3390/plants13202888 (PMC11511212; doi:10.3390/plants13202888)
Supplement: Supplementary file 1 [file plants-13-02888-s001.zip › plants-3157378-supplementary.pdf]

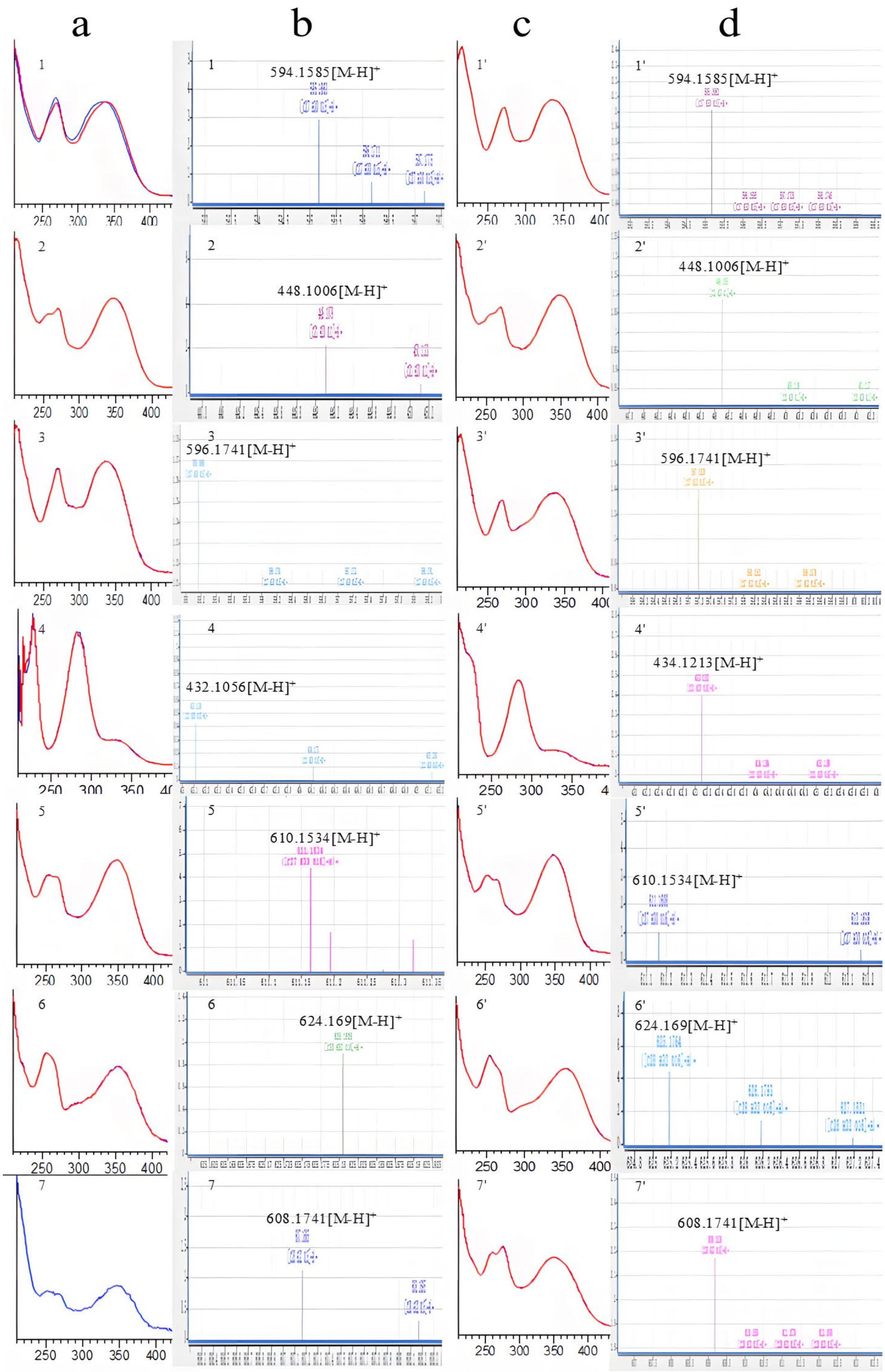

Figure S1. The UV and MS spectra of determined flavonoids in standards and samples prepared from lemon peels. line 1: vicenin-2; line 2: isoorientin; line 3: eriocitrin; line 4: vitexin; line 5: rutin; line 6: narcissoside; line 7:

diosmin; a: UV spectrum of sample; b: MS spectrum of sample; c: UV spectrum of standard; d: MS spectrum of standard..

Table S1. Summary of sequencing data of lemon leaves and information of clean reads.

Sample ID	Read Sum	Base Sum(G)	Q20%	Q30%	GC content%
CK1	33771220	10.02609143	97.86	93.82	44.57
CK2	30926264	9.156272186	97.95	94.06	44.7
CK3	35738712	10.64615934	97.73	93.5	44.63
SH1	29929866	8.910762508	97.72	93.53	44.67
SH2	25325271	7.544782142	97.84	93.78	44.61
SH3	33903482	10.11317148	97.72	93.49	44.31

Table S2. Unigene annotated statistics.

Annotated Database	Annotated_Number	300<=length<1000	length>=1000
KEGG_Annotation	15046(33.8%)	5016	6648
All_Annotated	44533(100.0%)	15695	16415

Table S3. Characterization of seven flavonoids from the extract of lemon peels by HPLC-DAD/ESIMS.

Peak No.	Standard							Sample				
	RT (min)	Compound Name	Molecular Formula	UV λmax (nm)	Theoretical Exact Mass (Da)	Mean Measured Mass (Da)	Mass Accuracy (ppm)	RT (min)	UV λmax (nm)	Theoretical Exact Mass (Da)	Mean Measured Mass (Da)	Mass Accuracy (ppm)
1	9.7611	vicenin-2	$C_{27}H_{30}O_{15}$	340	595.1667	594.1585	2.93	11.115	343	595.1662	594.1585	0.89
2	11.668	isoorientin	$C_{21}H_{20}O_{11}$	350	449.1083	448.1006	1.5	12.194	348	449.1085	448.1006	0.29
3	17.09	eriocitrin	$C_{27}H_{32}O_{15}$	341	597.1819	596.1741	1.02	18.703	339	597.182	596.1741	0.62
4	19.677	vitexin	$C_{21}H_{20}O_{10}$	274	435.1289	434.1213	0.83	19.927	271	433.1132	432.1056	0.61
5	21.501	rutin	$C_{27}H_{30}O_{16}$	349	611.1619	610.1534	0.5	23.592	349	611.1608	610.1534	0.16
6	37.171	narcissoside	$C_{28}H_{32}O_{16}$	350	625.1771	624.169	1.8	37.703	350	625.1764	624.169	0.06
7	43.323	diosmin	$C_{28}H_{32}O_{15}$	350	609.1825	608.1741	1.93	43.076	351	609.1819	608.1741	0.68

Table S4. The contents of 7 flavonoids in samples extracted from lemon peels treated or untreated with HP by HPLC-DAD. Data were presented as means ± standard deviation (n = 3).

Analyte	Nov-21		Feb-22		Oct-22	
	CK	SH	CK	SH	CK	SH
vicenin-2	0.14±0.123	0.467±0.335	0.066±0.014	0.031±0.01	0.075±0.033	0.086±0.037
isoorientin	0.117±0.154	0.504±0.276	0.059±0.006	0.034±0.007	0.133±0.048	0.105±0.111
eriocitrin	1.36±0.878	3.088±1.511	0.662±0.156	0.411±0.172	0.881±0.252	0.903±0.685
vitexin	0.028±0.004	0.039±0.028	0.013±0.004	0.01±0.006	0.029±0.006	0.032±0.026
rutin	0.166±0.031	0.234±0.129	0.127±0.021	0.107±0.049	0.199±0.055	0.206±0.131
narcissoside	0.115±0.017	0.203±0.066	0.133±0.023	0.127±0.054	0.181±0.053	0.172±0.074
diosmin	0.066±0.006	0.137±0.011	0.047±0.006	0.039±0.017	0.063±0.023	0.08±0.051
total flavonoids	5.887±1.359	11.894±5.074	8.839±2.641	6.331±6.088	14.454±1.679	13.477±1.33

Table S5. Linear regression, equation precision of 8 flavonoids from extract of lemon peels treated FA, the RSD values (%) of precision, repeatability, stability and recovery rate.

Analyte	Linear regression	R^2	Linear range (mg/mL)	Precision (n = 6)	Repeatability (n=6)	Stability (n=5)	Recovery rate (n=5)
vicenin-2	y = 25705x - 69.4	0.9997	0.0103-0.4378	0.58%	0.39%	0.32%	114.18%
isoorientin	y = 34263x - 18.067	0.9997	0.0041-0.4402	0.49%	0.46%	0.60%	96.08%
eriocitrin	y = 4936.6x + 81.992	0.9995	0.0791-3.8264	0.57%	1.04%	0.54%	105.14%
vitexin	y = 81697x - 16.783	0.9999	0.0030-0.0486	1.14%	1.02%	1.97%	112.71%
rutin	y = 21901x - 26.279	0.9999	0.0287-0.2596	0.56%	0.59%	0.54%	91.35%
narcissoside	y = 22516x - 20.946	0.9998	0.0325-0.1988	1.00%	1.90%	1.02%	107.49%
diosmin	y = 30089x - 36.979	0.9999	0.0108-0.0807	0.50%	0.71%	2.30%	88.91%
total flavonoids	y = 2.066x + 0.0009	0.9995	0.0086-0.0206	0.11%	1.20%	1.88%	93.50%

Table S6. Analysis of proteins and expression of differential genes in the phenylpropane metabolic pathway.

Differential gene ID	CK1	CK2	CK3	SH1	SH2	SH3	KEGG number	Enzyme name
c20062.graph_c0	11.7	13.93	11.17	7.41	4.11	4.09	K09755	F5H (ferulate-5-hydroxylase)
c20389.graph_c0	2.16	1.88	2.22	3.33	3.77	4.05	K12356	glucosyltransferase
c32725.graph_c0	12.96	10.84	9.69	5.3	3.91	4.88	K09755	F5H (ferulate-5-hydroxylase)
c37216.graph_c0	5.78	4.05	7.61	9.42	23.7	8.79	K00430	peroxidase
c39812.graph_c0	2.07	1.07	1.72	3.27	2.83	3.67	K12356	glucosyltransferase
c45689.graph_c0	5.06	4.49	6.8	8.54	10.08	11.54	K13065	HCT (O-hydroxycinnamoyltransferase)
